# Reengineering Vital Registration and Statistics Systems

**Published:** 2004-12-15

**Authors:** Kiumarss Nasseri

**Affiliations:** Public Health Institute, Tri-Counties Cancer Surveillance Program, Cancer Center of Santa Barbara, Santa Barbara, Calif

## To the Editor:

In his timely essay, "Reengineering Vital Registration and Statistics Systems for the United States," Charles J. Rothwell raises important issues about the registration of vital events in the United States (1). There is no question about the value of taking advantage of electronic technology to improve the quality and increase the utility of the registration system. The partnership mentioned by Rothwell is a step in the right direction. Currently, only a small portion of the data collected on paper death certificates is captured in machine-readable format. The rest — including valuable address and occupation information — is lost forever for population-based research and public health purposes.

My primary concern, however, is about the issues that were not discussed by Rothwell. First, the paper death certificate is a document whose legality precedes its public health importance. Unless electronic documents are legally accepted, we must have the paper documents.

Second, some important data are "mutilated" when captured in machine-readable format. According to National Center for Health Statistics (NCHS) guidelines, the place of birth of the deceased is coded only to the 50 states, the U.S. territories, and a few foreign countries like Canada and Mexico. All the other nations of the world are grouped together as one code! Granted, individuals born in foreign lands (i.e., first-generation immigrants) do not make up a large segment of U.S. death certificates, but from a public health point of view, they are an important group. Some states defy NCHS guidelines and collect the exact place of death for each deceased person in machine-readable format, but the utility of these data is limited to the state collecting the information; national organizations are forced to accept NCHS format. Also, information on the place of birth of a decedent's parents is of major epidemiological value because it identifies second-generation immigrants. Some states collect this information, but following NCHS guidelines, they do not capture it in machine-readable format.

The issue of accuracy is significant. Technically, we can evaluate the accuracy of information only against an independent document. Yet we accept many data on official, technical, and research documents at face value without verification and use the data to draw important conclusions. Information on occupation, for example, can be complex. Most people change their occupation several times, and upon death, a simple choice of occupation may not seem so clear. Or, perhaps the next of kin wishes to "upgrade" the occupation of the deceased. "Retired" and "housewife" are the most common occupations given for men and women, despite what careers they may have pursued. Nevertheless, with proper instructions and smart algorithms, we can improve the process of collecting information on occupation so that it can be used effectively for research purposes.

My main point in writing this letter is to draw attention to some important details that might be lost during the process of upgrading the current vital registration system to an electronic one. I would also like to suggest that the partnership of the National Association of Public Health Statistics and Information System, NCHS, and Social Security Administration be expanded to include a fourth member that represents the consumer side of these data — epidemiologists or other public health officials who have closely worked with these data for many years and are intimately aware of their utility and shortcomings.
